# The price of equality: determinants of the convergence in delivery costs in Bangladesh

**DOI:** 10.3389/fpubh.2026.1747772

**Published:** 2026-03-25

**Authors:** Martin Fischer, Masum Ali, Sanjib Saha

**Affiliations:** 1 Health Economics Unit, Department of Clinical Science (Malmö) Lund University, Forum Medicum, Lund, Sweden; 2 Nutrition and Health Science, Laney Graduate School, Emory University, Atlanta, GA, United States

**Keywords:** Bangladesh, cesarean section, delivery expenditures, demographic and health survey, health inequality, maternal health financing, out-of-pocket expenditure

## Abstract

Out-of-pocket (OOP) expenditures for childbirth in Bangladesh have increased substantially, intensifying financial pressure on lower-income households. While wealthier groups historically bore higher delivery costs, recent trends indicate a narrowing gap in spending across socioeconomic strata. This study investigates the drivers of this convergence in delivery-related expenditures between 2014 and 2022, distinguishing between demographic shifts and changes in medical service utilization. We analyzed nationally representative data from the 2014 and 2022 Bangladesh Demographic and Health Surveys using a retrospective two-stage stratified sampling design. Inequality in expenditures was assessed using the concentration index, and decomposition analysis was applied to quantify the contribution of explanatory factors. Between 2014 and 2022, the concentration index for delivery expenditures declined from 0.37 to 0.20 (Δ = −0.17, *p* < 0.01), reflecting a significant reduction in wealth-related inequality. During this period, the average cost of delivery rose from 9,486 to 13,217 BDT, and the cesarean section (CS) rate nearly doubled from 24 to 46%. Decomposition analysis revealed that the decline in inequality was driven almost entirely by increased CS and institutional deliveries among poorer households. CS alone accounted for 69% of the reduction in the concentration index, primarily due to increased uptake among less affluent groups. Normal institutional deliveries explained most of the remaining change, while maternal risk factors, antenatal care, and sociodemographic variables had minimal influence. These findings show that delivery expenditures have become more evenly distributed across wealth groups, driven primarily by changes in delivery mode rather than shifts in health or demographic risk. However, the sharp rise in cesarean sections as the major contributing factor and especially among poorer households—well above international recommendations—points to potential over-medicalization and increased financial vulnerability, with implications for health equity and system sustainability. In the absence of universal health coverage and given substantial out-of-pocket payments, greater spending equity may signal worsening financial protection, even as access and utilization become more equitable.

## 1 Introduction

The rising costs of delivery services in Bangladesh have become a significant financial burden for many households, particularly those in lower income groups. In recent decades, out-of-pocket (OOP) expenditure on healthcare, including delivery costs, have increased dramatically. In Bangladesh, OOP payments constitute a major share of total health expenditures, rising from 55.9% in 1997 to 63.3% in 2012, with delivery-related costs accounting for a substantial portion of this spending (1, 2). Given that healthcare financing in Bangladesh relies heavily on OOP payments, these rising costs pose a severe poverty risk, especially since most of the population belongs to lower income groups who pay these expenses directly without effective risk-pooling mechanisms (3).

Historically, in the early 2000s, higher delivery expenditures were predominantly borne by high-income groups, reflecting disparities in access to and utilization of healthcare services. However, over time, this gap has narrowed as delivery costs have risen across the income distribution. This convergence in expenditure levels is concerning because it implies that lower income households now face financial pressures comparable to wealthier groups, increasing their vulnerability to catastrophic health expenditures and impoverishment mechanisms (1–3).

This increasing financial burden is mirrored by a dramatic rise in CS rates in Bangladesh. From a mere 3.5% in 2004, CS rates surged to 33% in 2017–18 (4) and continue to rise, surpassing the World Health Organization’s recommended rate of 10–15% for all income groups (5, 6). Initially, higher CS rates were mostly observed in wealthier populations, but recent data indicate that lower income groups have also experienced significant increases (7). While this trend was previously interpreted as improved equitable access to obstetric care, the rapid escalation raises concerns about unnecessary CS procedures and associated economic burdens on poorer households. A single CS procedure could cost more than 4 months’ income for a typical household (2). Given its high cost and rapidly increasing prevalence, cesarean section is a strong candidate for the most influential factor shaping trends in rising overall delivery expenditures. Financial vulnerability is expected to be particularly acute for lower-middle income groups, which constitute most of the Bangladeshi population, according to the WHO poverty headcount criteria, with USD 8.30 per day. Consequently, especially poor families are forced to borrow money to cover CS costs. At the same time, the medical necessity of at least parts of the additional cesarean sections seems questionable. By 2022, CS rates across all wealth groups in Bangladesh exceeded WHO recommendations, with private healthcare facilities playing a dominant role in this increase (6).

The objective of this paper is to investigate the factors that have contributed to the convergence of delivery expenditure levels across different wealth groups in Bangladesh—a trend that, contrary to initial assumptions, signals increasing financial vulnerability among lower income households rather than improved equity health care. To interpret this pattern, it is useful to distinguish key equity dimensions in maternal care. Equity of access concerns whether women can reach facilities capable of safe delivery; equity of utilization captures whether interventions such as cesarean sections are used according to medical need; and financial risk protection reflects the burden of out-of-pocket costs for facility births and surgical deliveries (8, 9). These dimensions need not move together: increases in access to—and use of—facility-based and surgical births may occur alongside worsening financial protection in a system where most households must pay for cesarean sections themselves (10).

Specifically, this study aims to disentangle the relative contributions of shifts in demographic and risk factors (such as changes in maternal age, parity, and health status) from those related to medical service provision (including rising CS rates, healthcare commercialization, and changes in service pricing and availability) in driving the convergence of delivery expenditure across the wealth distribution. Although previous research has documented convergence in cesarean-section use and rising delivery expenditures in Bangladesh (2, 11, 12), no study has examined how these developments jointly shape out-of-pocket spending across the wealth distribution. This leaves unclear whether the observed convergence reflects improved access or a deterioration in financial protection or both. By integrating utilization trends with an economic-inequality perspective, our study fills this gap by decomposing the drivers of changing expenditure inequality between 2014 and 2022.

## 2 Materials and methods

### 2.1 Data sources and study population

We used data from the Bangladesh Demographic and Health Survey conducted in 2011 (13), 2017 (14), and (4), which are nationally representative and cross-sectional in design. The surveys were carried out to collect information related to sociodemographic, anthropometric, health and nutrition, by the National Institute for Population Research and Training of the Ministry of Health and Family Welfare in Bangladesh in collaboration with the ICF International (USA) and Mitra and Associates. The households were sampled using a two-stage stratified sampling procedure. In the first stage, the probability proportional procedure was followed for the Primary Sampling Unit (PSU), followed by a systematic selection procedure for the inclusion of the households in the second stage (13, 14). From the three surveys, we selected only the women for whom delivery information was available with no missing covariates, which are 95% of mothers with delivery information. Additionally, mothers from the regional division Mymensingh were excluded as the region did not exist in 2014. The exact sample selection process for this study is presented in Supplementary Figure S1.

### 2.2 Variables of interest

We included variables in three broad categories: (1) delivery-related, (2) pregnancy- and maternal health-related and (3) sociodemographic. The delivery-related variables were the cost of delivery, cesarian section and normal institutional delivery, both interacted whether the institution of birth was private or public. The cost of delivery is the dependent variable in this study. To ensure comparability of delivery costs across years, we adjusted all cost values to 2022 dollars using the consumer price index (CPI). The 2022 costs served as the reference and remained unadjusted. For prior years, we applied year-specific inflation factors: 1.48 for 2014 and 1.18 for 2017. These adjustment factors were derived from the World Bank CPI data sources (15). This standardization allowed for meaningful temporal comparisons by accounting for inflationary changes over the study period.

In terms of pregnancy and maternal health characteristics, we incorporated whether the infant was firstborn and whether the pregnancy was intentional. Antenatal care (ANC) visits were classified as fewer than 4 and 4 or more. The mother’s age was classified into two categories: teenage and older. The mothers’ health state was classified as underweight, overweight, or obese according to their body mass index (BMI) utilizing international thresholds. The sociodemographic factors include the educational attainment of the husband and mother, the employment status of the mother, and the wealth index. Furthermore, indicators for rural place of residence and regional division of residence were generated. BDHS computes the wealth index utilizing the standard DHS wealth index methodology via principal component analysis (14).

### 2.3 Statistical analysis

We are interested in explaining the determinants of changes in wealth-related inequalities for delivery expenditures between 2014 and 2022. We measure relative inequality in delivery-related expenditures $y_{\mathrm{it}}$ across the household wealth index $w_{\mathrm{it}}$ by the concentration index $C_{t}$ . When expenditures $y_{\mathrm{it}}$ are modeled by a linear additive regression model with explanatory variables $x_{\mathrm{ikt}}$

$$y_{\mathrm{it}}=\beta_{0}+\sum_{k}\beta_{k}\;x_{\mathrm{ikt}}+\epsilon_{\mathrm{ikt}}$$ (1)

the concentration index $C_{t}$ can be decomposed into an explained component and an unexplained residual component (16):

$$C_{t}=\sum_{k}(\frac{\beta_{k}\bar{x_{\mathrm{kt}}}}{\mu })C_{\mathrm{kt}}+\frac{GC_{\varepsilon }}{\mu },$$ (2)

where μ is the mean of expenditures $y_{\mathrm{it}}$ , the means $\bar{x_{\mathrm{kt}}}$ , and $C_{\mathrm{kt}}$ is the concentration index for the k-th regressor. The residual component reflects wealth-related inequality in expenditures that cannot be explained by systematic variation in the regressors across wealth groups.

Delivery expenditures are typically heavily right skewed, and unassisted home births include a sizable number of zeros. We, therefore, chose a nonlinear model to estimate Equation 1, applying a negative binomial regression model. To select among alternative generalized linear regression models such as Poisson regression and standard multiple linear regression, we relied on the Bayesian information criterion (BIC). However, the results for the expenditure estimation were not particularly sensitive to the specific choice of the generalized linear regression model. As the decomposition of the concentration index relies on a linear model, we apply a linear approximation to the nonlinear binomial regression model using the partial effects at means (17).

The DHS 2022 only covers costs for hospital births, including c-sections and normal births, but not for assisted home births. However, the costs for assisted home births are primarily determined by personal costs. To impute costs for home births in 2022, we first select relevant predictors for expenditures using a penalized lasso Poisson regression for all combinations of birth attendance personnel from the waves 2014 and 2017 as regressors. We predict costs for home births based on selected birth attendance personal combinations from the penalized regression for the year 2022. To evaluate the robustness of our imputation strategy, we compare the change in the concentration index based on imputed and actual costs between 2014 and 2017, as we have costs for all birth modes in those two survey years. Reassuringly, the change in concentration indices is essentially indistinguishable whether imputed costs or self-reported costs are being used, confirming our prior belief that homebirth costs are mainly determined by personnel.

To explain changes in wealth-related inequality in expenditures over time measured by the concentration index $C_{t}$ , we adapt the total differential approach by Wagstaff et al. (16) to Equation 2. The total differential allows us to investigate whether changes in the relative inequality in expenditures stem from changes in the regression coefficients $\beta_{x}$ from the expenditure model, the means $\bar{x_{\mathrm{kt}}}$ , or the concentration indices of the explanatory variables $x_{\mathrm{ikt}}$ . The change in $C_{t}$ can then be approximated locally by using Equation 3


$$\begin{array}{c} \begin{array}{l} \mathrm{dC}=-\frac{C}{\mu }\mathrm{d\alpha}+\sum_{k}\frac{\bar{x_{\mathrm{kt}}}}{\mu }(C_{\mathrm{kt}}-C)d\beta_{k}+\sum_{k}\frac{\beta_{k}}{\mu }(C_{\mathrm{kt}}-C)d\bar{x_{k}}+\\ \sum_{k}\frac{\beta_{k}\bar{x_{\mathrm{kt}}}}{\mu }dC_{k}+d\frac{GC_{\varepsilon }}{\mu } \end{array} \end{array}$$ (3)

For inference on each explanatory variable on the change in the concentration index, we calculate bootstrap confidence intervals using 1,000 bootstrap replications for stable 95-percentile bootstrap confidence intervals (18). As DHS data rely on survey weights, we apply bootstrap resampling methods designed for complex survey data (19).

### 2.4 Ethical considerations

The present study used publicly available, de-identified secondary data from the Bangladesh Demographic and Health Survey (BDHS). Formal ethical approval was not required because the data are publicly accessible and contain no identifiable information about the survey participants. Permission to use the BDHS data was obtained through the Demographic and Health Surveys (DHS) Program.^1^ All analyses were conducted in compliance with the DHS Program’s data use policy, which ensures participant anonymity and confidentiality.

## 3 Results

Table 1 presents descriptive statistics for the two waves: BDHS’14 and BDHS’22. The concentration index decreased from 0.37 to 0.20 (Δ = −0.17, *p* < 0.01), indicating a sizable reduction in inequality of expenditure. Delivery-related outcomes showed marked shifts: the mean cost of delivery rose substantially from BDT 9,486 to BDT 13,217 adjusted for CPI (Δ = 3,730, *p* < 0.01), whereas the proportion of cesarean sections nearly doubled (from 0.24 to 0.46, Δ = 0.22, *p* < 0.01), and normal institutional deliveries (NID) increased modestly (Δ = 0.04, <0.01), with most increases in CS and NIDs occurred in private facilities. Table 1 also reveals substantial significant changes across key maternal and socio-demographic indicators between 2014 and 2022.

**Table 1:** Descriptive statistics.

Variables	(1)	(2)	(3)	(4)
	2014	2022	Δ = (2)–(1)	*p*-value Δ
Concentration index				
Conc. index	0.37	0.20	−0.17	<0.01
Delivery				
Cost of delivery	9,486	13,217	3,730	<0.01
Cesarian section	0.24	0.46	0.22	<0.01
thereof private	0.18	0.39	0.20	<0.01
Normal institutional delivery	0.16	0.20	0.04	<0.01
thereof private	0.05	0.08	0.03	<0.01
Pregnancy and maternal health				
First born	0.40	0.38	−0.03	0.01
ANC (4 or more)	0.32	0.40	0.08	<0.01
ANC (1–3)	0.46	0.53	0.06	<0.01
Wanted pregnancy	0.10	0.07	−0.04	<0.01
Wanted later pregnancy	0.15	0.12	−0.03	<0.01
Mother underweight	0.25	0.15	−0.10	<0.01
Mother overweight	0.17	0.29	0.12	<0.01
Mother obese	0.03	0.05	0.02	<0.01
Mother age <20	0.21	0.14	−0.07	<0.01
Mother age ≥35	0.06	0.08	0.02	<0.01
Socio-demographics				
Husband high education	0.15	0.21	0.06	<0.01
Mother high education	0.11	0.19	0.08	<0.01
Mother working	0.22	0.21	−0.01	0.16
Living in rural area	0.68	0.66	−0.02	0.02
N	4,422	4,356	8,778	8,778

Columns 1 and 2 show the means by survey year. Column 3 gives the mean difference ∆ between and column 5 the *p*-value for testing if mean difference between both years is different from zero (H_0_: ∆ = 0). Distributions of regional divisions across waves are given in Supplementary Table S1. Source: BDHS 2014, 2022. Own calculations.

**Figure 1:**
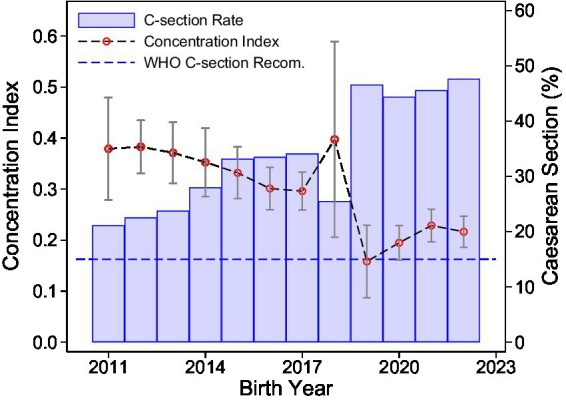
Inequality in health care expenditures and C-section rate. The figure shows the concentration index for costs of delivery and the rate of cesarian sections across birth cohorts from —2011 to 2022. The wealth index is used as the ranking variable for the concentration index. The blue dashed line represents the upper range of the WHO recommended rate of cesarean sections of 15% of all births. Source: BDHS 2014, 2017, and 2022, Bangladesh. Own calculations.

Figure 1 illustrates the trends in both the concentration index for delivery costs and the rate of CS deliveries in Bangladesh across birth cohorts from 2011 to 2022. Over this period, the CS rate rose sharply, surpassing the World Health Organization’s recommended upper threshold of 15% and reaching approximately 45% by 2022. In parallel, the concentration index, which measures inequality in delivery costs by wealth, declined steadily from approximately 0.37 in 2014 to 0.22 in 2022, indicating a reduction in wealth-related disparities in delivery expenditures.

**Figure 2:**
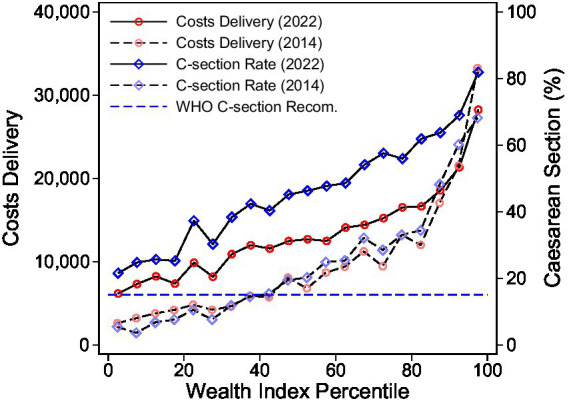
Health care expenditures and C-section rate. The figure shows the mean costs of delivery and the mean rate of cesarean sections along percentiles of the wealth index. The blue dashed line represents the upper range of the WHO recommended rate of cesarean sections of 15% of all births. The costs of delivery are measured in BDT. The data are weighed by survey weights. Source: BDHS 2014 and 2022, Bangladesh. Own calculations.

Figure 2 presents the distribution of mean delivery costs and CS rates across wealth index percentiles in Bangladesh for the years 2014 and 2022. The results reveal a pronounced positive gradient: both delivery costs and CS rates increase steadily with increasing household wealth. Rising expenditures in all but the highest wealth groups between 2014 and 2022 lead to a shrinking wealth gap. Similarly, the CS rate sharply increased across the wealth distribution except for the wealthiest household, which already experienced a very high rate of over 40% in 2014 and has since been stable. By 2022, all households exceed the World Health Organization’s recommended maximum CS rate of 15%.

The results in Table 2 present the marginal effects from the negative binomial regression of various factors on delivery-related expenditures for the years 2014 and 2022. Cesarean section deliveries were associated with the largest increase in expenditures in both years, although the marginal effect for CS in public facilities only increased marginally from 23,836 in 2014 to 24,859 in 2022. In both years there is a moderate but significant price increase for CS in private facilities for both years (1,793 and 1,326). In contrast, normal institutional deliveries saw a substantial rise in associated expenditure, with the marginal effect nearly doubling from 6,845 to 12,403over the same period for public facilities. The same is observed for the price premium for vaginal birth in private facilities which had additional price increases from 2,986 to 5,710.

**Table 2:** Marginal effects for expenditures.

Variables	Year 2014		Year 2022	
	Marg. effect		Marg. effect	
Cesarian section	23,836	(1203)	24,859	(934)
× private institution	1793	(315)	1,326	(402)
Normal institutional delivery	6,845	(606)	12,403	(1114)
× private institution	2,986	(621)	5,710	(1067)
First born	290	(130)	509	(211)
ANC (4 or more)	761	(156)	1,270	(357)
ANC (1–3)	187	(115)	614	(290)
Wanted pregnancy	−81	(140)	−210	(347)
Wanted later pregnancy	−262	(138)	330	(407)
Husband high education	918	(218)	530	(277)
Mother high education	337	(239)	1,088	(289)
Mother Working	−134	(108)	−14	(272)
Mother underweight	−152	(109)	119	(287)
Mother overweight	499	(185)	−126	(223)
Mother obese	624	(694)	1,292	(470)
Mother Age <20	26	(145)	−336	(288)
Mother Age ≥35	−213	(169)	−196	(258)
Living in rural area	101	(113)	−364	(226)
Division: Barisal	−216	(186)	243	(391)
Division: Chittagong	395	(194)	141	(325)
Division: Dhaka	−593	(168)	−408	(342)
Division: Khulna	−1,132	(147)	−1726	(292)
Division: Rajshahi	−925	(145)	−2,138	(273)
Division: Rangpur	−723	(178)	−1,616	(290)
Observations	4,422		4,356	

Estimates show marginal effects at means from negative binomial regression. Sylhet is omitted regional division. Standard errors are in parentheses. The estimates are weighted via BDHS survey weights. Source: BDHS 2014, 2022. Own calculations.

The total differential decomposition of the change in inequality from 2014 to 2022, as presented in Table 3, indicates that the overall concentration index declined by 0.17. In line with Figure 2, the largest contributor to this reduction was cesarean section deliveries, accounting for a decrease of 0.116 in the concentration index (−69% of the total change). Of this total effect, 4 percentage points stems from an interaction effect of giving birth via CS in a private hospital. Separating the total effect of cesarean section into its components, almost the entire effect can be attributed to the shift in the concentration of c-sections to poorer families ( $C_{x}$ ). Normal institutional deliveries also contributed notably to the decrease in the concentration index, with a reduction of 0.044 (−25%). While the contribution from normal institutional deliveries stems partially from a shift to less wealthy families, changes in the regression coefficients for expenditures ( $\beta_{x}$ ), i.e., the prices of non-Cesarean section institutional deliveries, explained roughly half of the effect. The interaction effect from private institutions explains 2 percentages points of the total effect from NIDs.

**Table 3:** Total differential decomposition of change in inequality, 2014–2022.

Variable	(1)	(2)	(3)	(4)	(5)
	$\beta_{x}$	$\beta_{x}$	$CI_{x}$	$\text{Tota}l_{x}$	Δ %
C-section	0.000	0.010	−0.121	−0.110	−65
× private institution	−0.000	0.002	−0.008	−0.006	−4
Normal institutional delivery	−0.018	−0.008	−0.013	−0.040	−23
× private institution	−0.001	−0.001	−0.002	−0.004	−2
First Birth	−0.002	0.000	−0.001	−0.003	−2
ANC (4 or more)	−0.002	−0.001	−0.001	−0.003	−2
ANC (1–3)	−0.008	−0.000	−0.001	−0.009	−6
Wanted pregnancy	0.001	−0.000	−0.000	0.001	0
Wanted later pregnancy	−0.004	−0.000	−0.000	−0.005	−3
Mother underweight	−0.004	−0.001	−0.000	−0.005	−3
Mother overweight	−0.000	0.000	−0.002	−0.002	−1
Mother obese	0.000	0.000	−0.000	0.000	0
Maternal Age <20	0.004	0.000	−0.000	0.004	2
Maternal Age ≥35	−0.000	0.000	−0.000	0.000	0
Husband high education	−0.001	0.001	−0.001	−0.001	−1
Mother high education	0.001	0.000	−0.000	0.001	1
Mother Working	−0.001	−0.000	0.000	−0.002	−1
Living in rural area	0.019	0.000	0.000	0.019	11
Division: Barisal	−0.002	0.000	0.000	−0.001	−1
Division: Chittagong	0.002	−0.000	−0.001	−0.000	0
Division: Dhaka	−0.002	−0.001	−0.003	−0.006	−4
Division: Khulna	0.002	0.002	−0.001	0.002	1
Division: Rajshahi	0.006	0.001	−0.001	0.006	4
Division: Rangpur	0.005	0.001	0.001	0.007	4
Residual				−0.012	−7
Total Δ CI	−0.005	0.004	−0.156	−0.169	100

Column 1 gives changes in the change in the concentration index attributed to changes in the partial effects or the specific regressor. Column 2 shows changes attributed to level changes in each covariate. Column 3 shows the changes in the concentration of X . The total change in all three parts combined is given in column 4 and, in relative terms, in column 5. The residual term reflects part of the change in the CI not captured by X and the regression modeling. Source: BDHS. Own calculations.

**Figure 3:**
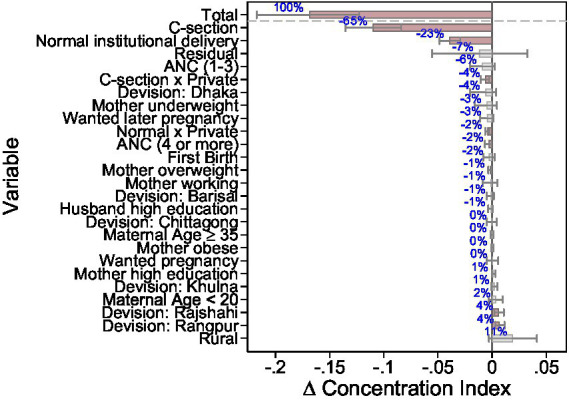
Relative contribution to total differential decomposition of change in wealth-related expenditure inequality, 2014–2022. The figure shows the relative contribution of predictors of delivery expenditures. 95% bootstrap confidence intervals were added using 1,000 bootstrap replications. The data are weighted by survey weights. Statistically significant contributors on the 5% are colored in red. Source: BDHS2014 and 2022, Bangladesh. Own calculations.

Factors such as antenatal care with one to three visits (−6%), four or more visits (−2%), maternal underweight (−3%), and wanted later pregnancies (−1%) made modest negative contributions. Further demographic variables, including first birth, wanted pregnancy, maternal obesity, maternal age ≥35, mothers’ high education, and maternal employment, had negligible or no effect on the overall change. In contrast to the large negative contributions from delivery-mode variables, rural residence contributed positively to the CI change (+0.019; ~11% of the total), indicating a modest increase in pro-wealth concentration associated with changes in the rural expenditure gradient. Similarly, the more rural divisions Rajshahi (+0.006) and Rangpur (+0.007) likewise slightly raised pro- wealth inequality, with effects driven predominantly by changes in coefficients (Δβ) rather than by compositional shifts or changes in the socioeconomic ranking of these covariates. The positive contribution of rural residence indicates that this factor slowed the overall convergence in expenditure inequality, although its effect was modest relative to the large negative contributions from delivery-mode variables. This is further underlined by Figure 3, which displays the relative contributions of various factors to the total differential decomposition of the change in wealth-related inequality in delivery expenditures in Bangladesh from 2014 to 2022 together with their 95% CI. Apart from cesarean section, institutional deliveries and their interaction with private ownership, only antenatal care visits and the two more rural divisions Rajshahi and Rangpur were statistically significant factors in determining the change in wealth-related expenditure inequalities at the 5% significance level, with their CI excluding zero.

## 4 Discussion

In this study, we estimated the change in inequality in the cost of delivery and the factors that explain it using nationally representative surveys for Bangladesh. Health care expenses for delivery between the two survey waves, 2014 and 2022, became more equally distributed across the wealth distribution. This transition is almost exclusively driven by alterations in the delivery method in less wealthier households, shifting home births to institutional deliveries with and without cesarean section. Among all delivery-related factors, the rise in cesarean section rates clearly dominates, explaining the vast majority of the shift in expenditure inequality. Changes in risk factors or sociodemographic variables play no significant role in explaining the shift. We will discuss the clinical, economic and policy implications of our findings.

First, we interpret our descriptive findings on the changes in the mode of delivery within a broader clinical context. For CS, the major driver of the change in the equality of delivery expenditures, our estimates show that the WHO-recommended CS rate is exceeded for all wealth groups by 2022. While there is debate concerning the WHO CS rate as an admissible target, recent meta-analysis indicated no survival advantage for mothers or children when CS rates exceeded this threshold (20). The very elevated rate of cesarean sections suggests potentially superfluous medical intervention. Some degree of unnecessary treatment is also corroborated by our finding that changes in maternal risk factors do not contribute to the observed changes in delivery expenditure. This problem is potentially even more severe given recent evidence for simultaneous under- and overtreatment, as there is no guarantee even in the presence of overall high CS rates that all pregnant women in need of medical treatment (4). We note that in the absence of actual clinical data on the necessity of CS in the DHS our assessment of excess medical treatment is indirect and needs caution. A systematic evaluation of whether the increase in cesarean sections reflects clinical need lies beyond the scope of this study and cannot be conducted reliably with DHS data.

Secondly, the economic implications of rising CS rates for less wealthy households in Bangladesh extend beyond clinical outcomes by putting significant strain on household financials. Paradoxically, more equitable access to and spending for medical care likely increases the risk of poverty in Bangladesh. Socioeconomic inequalities in CS rates across household wealth were previously partially framed as a problem of unequal access to medical care. Focusing on health care expenditures, which are predominantly out of pocket, we demonstrate that achieving a more equal distribution of CS by increasing access likely leads to an increase in poverty risk among vulnerable households in low- to middle-income countries such as Bangladesh.

Putting delivery costs into perspective, for the wealthiest households, CS costs constitute approximately 2.7% of annual income. Equivalent expenses could consume over half of the poorest quintile’s earnings, exacerbating financial vulnerabilities in low-income populations. As of 2025, the average monthly income ranges from 26,100 BDT (218.54 USD) for general workers to 8,000 BDT (66.99 USD) for minimum wage earners (21). In contrast, CS deliveries cost approximately 21,506 BDT (181 USD) in public facilities and 612 USD in private facilities (4), consuming 83–913% of the monthly income depending on socioeconomic status. Normal vaginal deliveries remain more affordable at 3,565 BDT (30 USD) (2) but still represent 14–45% of the monthly earnings for low-income households. This cost burden forces 79% of families to borrow money or liquidate assets for delivery expenses (22). A further structural constraint exacerbating this vulnerability is the near-absence of health insurance coverage in Bangladesh. Across BDHS waves, fewer than 1% of respondents report using insurance to finance delivery care, consistent with national evidence indicating extremely limited formal risk-pooling mechanisms (23). Thirdly, we explore the healthcare policy implications of our findings. The mechanisms driving the disparities intertwine supply-side incentives and demand-side perceptions. Providers in Bangladesh’s predominantly private healthcare system (accounting for 66% of CS procedures) face reimbursement structures that favor surgical deliveries, particularly when serving wealthier clients who can afford premium rates. Concurrently, upper-income groups increasingly perceive CS as safer and more modern—a trend documented in other LMICs such as Brazil (24) and Egypt (25)—while public sector subsidies fail to adequately offset costs for poorer households. These dynamics create an institutional reinforcement cycle where private facilities cater to affluent demand for elective CS, whereas cost barriers persist for medically necessary procedures in vulnerable populations. These findings underscore that inequality mechanisms depend on health system architectures. Bangladesh’s hybrid public–private model—while reducing absolute poverty-related access gaps—created new inequality vectors through unregulated private sector pricing.

Interestingly, while private facilities charge higher fees on average, supplementary analysis of our BDHS data shows that this price premium is concentrated among higher-income households, with relatively limited price discrimination across most of the wealth distribution. As a result, the expansion of private-sector provision contributes only modestly to the observed convergence in expenditures; the primary driver remains the increased uptake of cesarean sections among lower-income households at broadly similar prices. These findings have direct implications for Bangladesh’s progress towards Universal Health Coverage (UHC). UHC requires that all individuals can access needed health services without suffering financial hardship—a commitment embedded in SDG target 3.8 (26). In Bangladesh, where over two-thirds of health spending is paid out-of-pocket and public risk-pooling mechanisms remain limited (27), greater equality in spending may paradoxically indicate that poorer families now face a heavier financial burden from surgical deliveries. Previous projections have warned that catastrophic and impoverishing health expenditures in Bangladesh are likely to increase rather than decline by 2030 if current financing trends persist (28). Our decomposition results support this concern: the narrowing expenditure gap is not the product of improved financial protection but of a shift towards costlier delivery modes among the least affluent. Achieving meaningful progress towards UHC in maternal health will therefore require not only expanding access to facility-based care but also strengthening prepayment and risk-pooling mechanisms that shield households, especially poorer ones from the financial consequences of medically unnecessary CS.

A limitation of the study is the duration of the recollection period. In the BDHS, the recall time is 5 years, which is excessively lengthy for accurately reporting all occurrences linked with cesarean births and their accompanying costs. Furthermore, information regarding the necessity of CS or how the clinical status of the patients may warrant the procedure is missing, which does not allow a direct assessment of potential overtreatment. Additional data on this matter could have reinforced our interpretation. A comprehensive cost assessment associated with healthcare professionals, including doctors and nurses, for various activities related to CS (patient care, administrative duties, personal time, opportunity cost, and laboratory expenses) could not be conducted because of a lack of available information on these matters.

Methodologically, our use of the concentration index (CI) and its decomposition involve several limitations. First, the decomposition relies on marginal effects derived from a negative binomial expenditure model, which constitutes a linear approximation to a nonlinear regression model. While such approximations can introduce error, the close alignment between the observed change in the CI and the sum of the decomposed contributions - reflected in a residual of only 7% - indicates that the approximation is sufficiently accurate for this application. Second, the CI measures inequality in terms of the relative socioeconomic ranking of households rather than their absolute financial burden. This makes it well suited for assessing convergence in wealth-related inequality over time, which is the primary focus of our analysis. However, it does not capture the magnitude of the economic strain imposed on households. A key strength of this study is its use of the most recent data to illustrate the continuously increasing prevalence of cesarean sections and delivery expenditures, complemented by earlier nationally representative datasets to examine long-term trends. Nationally representative surveys such as the Demographic and Health Surveys (DHS) remain the most comprehensive and widely utilized sources for population-level data on cesarean births.

## 5 Conclusion

This study reveals that delivery-related healthcare expenditures have become more evenly distributed across wealth groups, driven mainly by a shift from home- to facility-based births rather than changes in risk factors or demographics. Cesarean sections are the primary contributors to the distributional shift in costs. The very high number of cesarean sections raises concerns about potential overuse. More equitable spending does not necessarily reflect improvements in access to needed healthcare. Rather, the high costs and increased usage of cesarean sections place a substantial financial burden on all households, increasing the risk of poverty, particularly among the poor. Policy responses should focus on balancing access with clinical appropriateness. Measures such as progressive pricing to deter unnecessary procedures among wealthier groups, stricter regulation of private providers, and real-time monitoring of CS rates by socioeconomic status could help address economically motivated overuse. Policy lessons emerge from Thailand’s universal CS coverage under national insurance (29), suggesting that Bangladesh could mitigate disparities by standardizing private facility charges while expanding public-sector surgical capacity. For Bangladesh, our findings suggest that it is advisable to implement public health policies that regulate unnecessary cesarean use and to expand pooled prepayment schemes that protect households from the financial burden of medically indicated facility births. However, although the extremely high cesarean section rates clearly point to substantial overuse, future research should more rigorously assess both necessity and potential overuse using clinically richer data sources than the DHS. This is particularly important given the possibility of simultaneous under- and overtreatment.

## Data Availability

The datasets presented in this study can be found in online repositories. The names of the repository/repositories and accession number(s) can be found at: https://www.dhsprogram.com/data/dataset/Bangladesh_Standard-DHS_2022.cfm?flag=0.

## Footnotes

1
https://dhsprogram.com
