# Supplementary material for: The price of equality: determinants of the convergence in delivery costs in Bangladesh
Source: Front Public Health. 2026 Mar 25;14:1747772. doi: 10.3389/fpubh.2026.1747772 (PMC13057423; doi:10.3389/fpubh.2026.1747772)
Supplement: Supplementary file 1 [file Data_Sheet_1.pdf]

Supplementary Tables and Figures

Sample Selection and Data

Figure S1 shows the different sample selection criteria for our final analysis sample. We use the wave 2017 only for supplementary analysis. We drop individuals if they have one or more missing values for covariates or the dependent variable of medical expenditures at birth. 5-7% of individuals have one or missing values in covariates. Most missing values stem from missing information on ANC. We could not detect any substantial association of missing values with our ranking variable household wealth for the decomposition.

The wave in 2022 had two additional challenges: First, expenditures on home births were not collected. We estimated expenditures on home births based on costs for attending personnel in earlier waves and predicted costs as described in the method section. Additionally, the BDHS did not collect anthropometric measures for 50% of the sample due to Covid. Instead of simply dropping those individuals completely, we imputed BMI, after first assessing the plausibility of a missing-completely-at-random mechanism. For this we regressed a missing-BMI indicator on the full set of covariates. The p-value for the joint F-test was 0.99, indicating no detectable systematic pattern in missingness. Based on this, we proceeded with the imputation to retain sample size and precision. As additional sensitivity analysis we also dropped the individuals with missing BMI in 2022 and run the analysis without imputed data. The results of for the relative contribution in our decomposition are almost identical with the main results as in Supplementary Figure S2 and clearly within the margin of error.

Figure S1: Sample size selection process

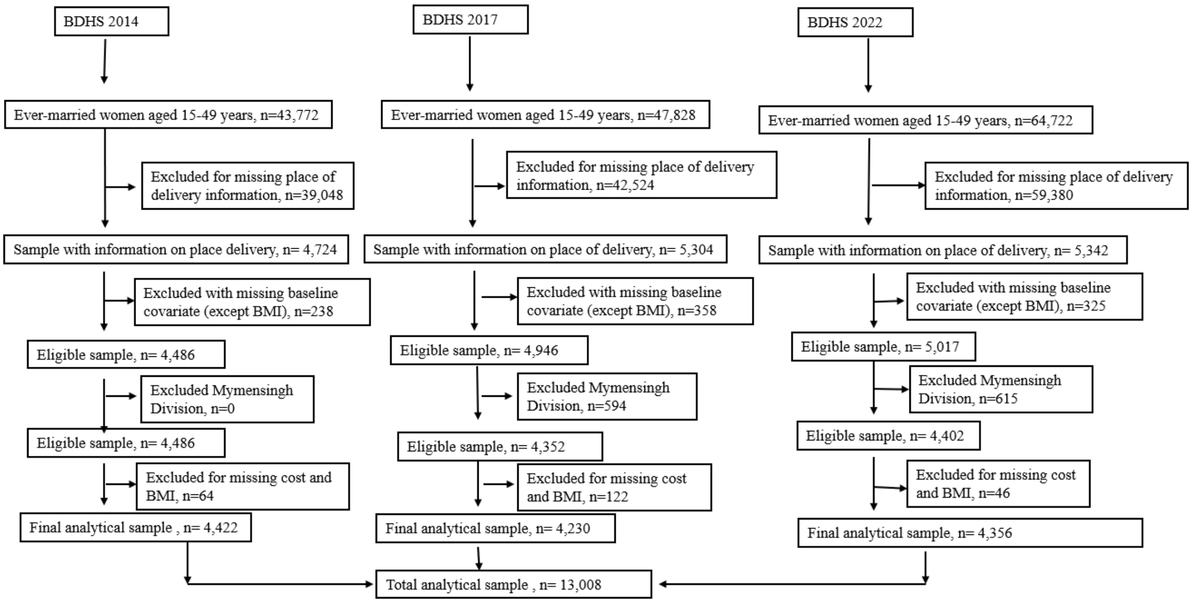

Figure 1: Study sample size selection procedure

Notes: The figure gives a flowchart showing our sample selection criteria for the three BHDS waves used in the analysis. The main analysis is based upon BHDS 2014 and 2022. BHDS 2017 is only used for estimating a model for homebirth in 2022 and is not part of the main analysis.

Figure S2: Decomposition if missing BMI in 2022 dropped

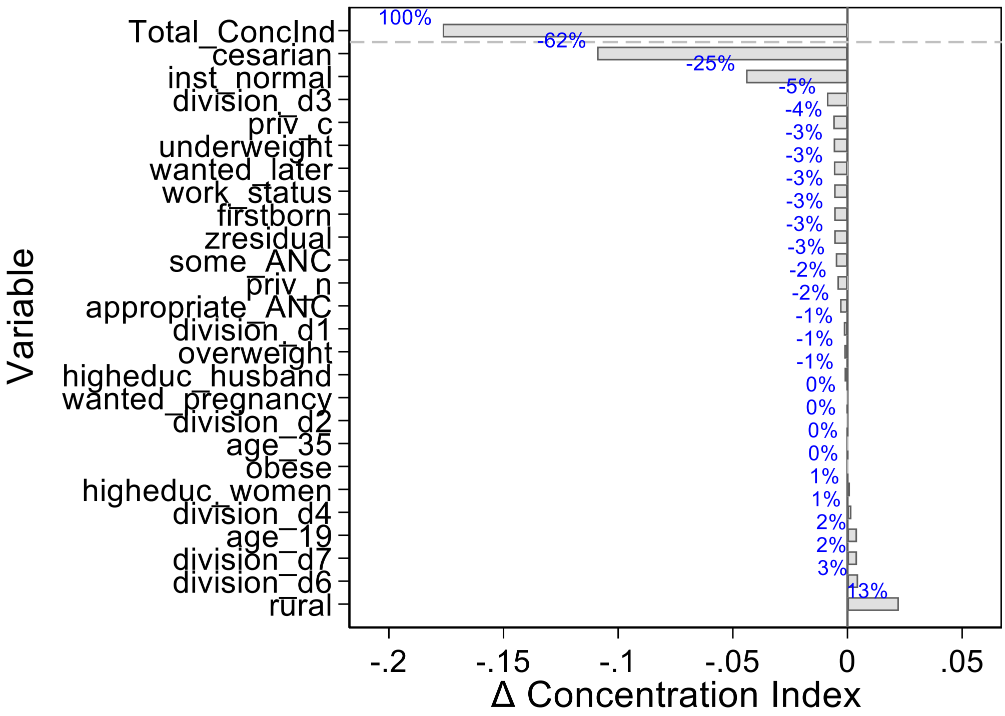

Notes: Replicates Figure 3 *Relative contribution to total differential decomposition of change in wealth-related expenditure inequality, 2014–2022* dropping all mothers with missing andromorphic variables in 2022. This reduces the sample size in 2022 by 50% but representative is preserved as missing values are MCAR.

Supplementary Table S1: Additional Descriptive Statistics

	(1) 2014	(2) 2022	(3) Δ = (2)-(1)	(4) *p*-value Δ
**DEVISIONS:**				
Barisal	0.12	0.12	0.00	0.53
Chittagong	0.19	0.20	0.01	0.50
Dhaka	0.18	0.17	-0.01	0.29
Khulna	0.12	0.13	0.01	0.10
Rajshahi	0.12	0.11	-0.01	0.33
Rangpur	0.12	0.13	0.01	0.13
Sylhet	0.15	0.13	-0.02	0.02
**N**	4,422	4,356	8,778	8,778

Notes: Columns 1 and 2 show the means by survey year. Column 3 gives the mean difference Δ between and column 5 the p-value for testing if mean difference between both years is different from zero (H_0: Δ = 0). *Source:* BDHS 2014, 2022. Own calculations.

Differential Pricing

A supplementary analysis of BDHS delivery-cost data shows little evidence that price discrimination across the wealth distribution contributes to the observed convergence in out-of-pocket expenditures. Although private facilities charge higher fees on average, this price premium is concentrated among the wealthiest households, while absolute prices for both cesarean and normal institutional deliveries remain broadly stable across most of the income distribution, as illustrated in Supplementary Figure S3 and S4. In lower- and middle-income groups, private and public fees differ only modestly, implying that changes in pricing structures cannot explain the narrowing of wealth-related inequality. Instead, the convergence is driven overwhelmingly by rising cesarean section use among poorer households at relatively uniform price levels. In effect, private facilities increase revenue primarily through higher **volume** rather than higher **prices**, meaning that utilization patterns—not price discrimination—account for the reduction in the concentration index.

Appendix Figure S3: Change in CS Rate and Price Premium of CS along HH Wealth

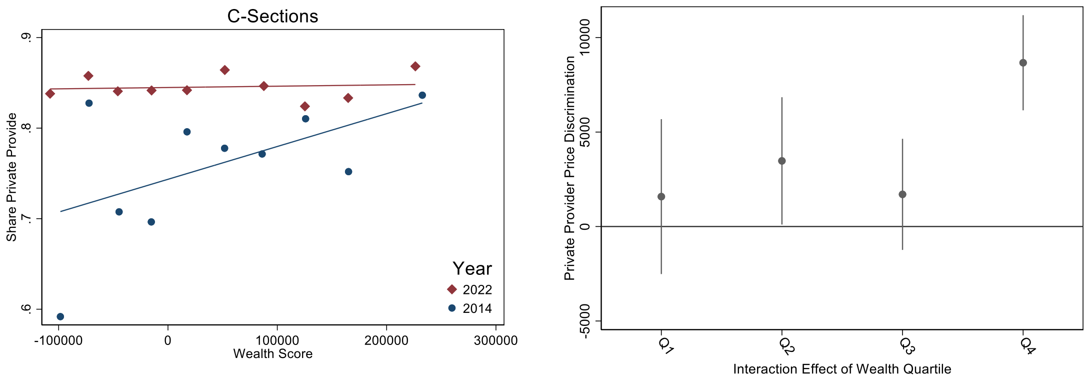

Notes: Left figure plots CS rate by year along wealth index. The right figure interaction effects of CS on expenditure by wealth quartiles from a linear regression. *Source:* BDHS 2014, 2022. Own calculations.

Appendix Figure S4: Expenditure by Mode of Delivery

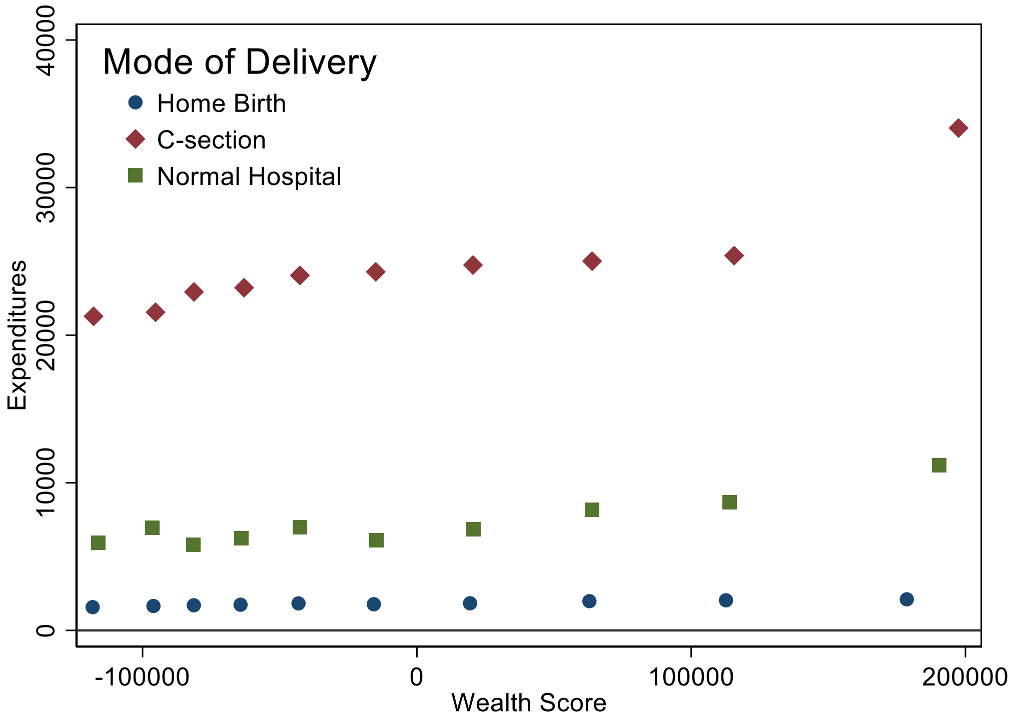

Notes: Left figure plots expenditures by birth mode along wealth index. The right figure plots expenditure data for CS as a series of marks against a single magnitude axis. Box Plot is added. Expenditure outliers are top censored at 100,000 for better visualization.
