# Supplementary material for: The price of equality: determinants of the convergence in delivery costs in Bangladesh
Source: Front Public Health. 2026 Mar 25;14:1747772. doi: 10.3389/fpubh.2026.1747772 (PMC13057423; doi:10.3389/fpubh.2026.1747772)
Supplement: Supplementary file 2 [file Data_Sheet_2.pdf]

Technical Appendix: The Price of Equality: Determinants of the Convergence in Delivery Costs in Bangladesh

Bootstrap Inference - Decomposition Confidence Intervals

Outcome, Socioeconomic Rank, and Survey Weights

Let y_i denote the health outcome for individual i in survey year t, and let r_i denote the individual's fractional rank in the distribution of socioeconomic status. All descriptive and model-based quantities are computed using the survey sampling weights w_i, which reflect unequal probabilities of selection and ensure that estimates represent the target population. Weighted means are defined as

$$\bar{z} = \frac{\sum_i w_i z_i}{\sum_i w_i},$$

and weighted covariances as

$$\mathrm{Cov}(z_i, r_i) = \frac{\sum_i w_i (z_i - \bar{z})(r_i - \bar{r})}{\sum_i w_i}.$$

Concentration Index

Socioeconomic inequality in the outcome is summarized using the Wagstaff concentration index,

$$CI_y = \frac{2}{\bar{y}} \mathrm{Cov}(y_i, r_i),$$

which is negative when the outcome is concentrated among the poor and positive when concentrated among the rich. Fractional ranks r_i are computed within each survey year using the weighted cumulative distribution of the socioeconomic variable.

Nonlinear Outcome Model

To relate the outcome to its determinants, we estimate a nonlinear model of the form

$$\mathbb{E}(y_i \mid X_i) = g(X_i \beta),$$

where $g(\cdot)$ is the mean function of a negative binomial regression model and X_i is a vector of covariates. The model is estimated using probability weights w_i, yielding consistent estimates of the conditional mean under standard survey-weighted M-estimation theory. For each covariate x_{ij}, we compute the marginal effect

$$\frac{\partial \bar{y}}{\partial x_j},$$

evaluated at sample means. These marginal effects are used to construct elasticities.

Decomposition of the Concentration Index

Following Wagstaff et al. (2003), the concentration index can be decomposed as

$$CI_y = \sum_j \eta_j \, CI_{x_j} + \frac{GC_\varepsilon}{\bar{y}},$$

where

$$\eta_j = \frac{\partial \bar{y}}{\partial x_j} \cdot \frac{\bar{x}_j}{\bar{y}}$$

is the elasticity of the outcome with respect to covariate x_j, and

$$CI_{x_j} = \frac{2}{\bar{x}_j} \operatorname{Cov}(x_{ij}, r_i)$$

is the concentration index of x_j. The contribution of covariate x_j to overall inequality is

$$C_j = \eta_j \, CI_{x_j},$$

and the share of inequality explained by x_j is C_j / CI_y.

Bootstrap Inference

Analytical standard errors for the decomposition components are not available in closed form for nonlinear models. We therefore rely on a nonparametric bootstrap that resamples the entire estimation procedure.

We generate B sets of bootstrap replicate weights $\{w_i^{(b)}\}_{b=1}^{B}$ using the Rao–Wu rescaling bootstrap. Each set of replicate weights corresponds to a bootstrap pseudo-sample that preserves the original survey weights as importance weights. For each bootstrap replicate b, we:

1. compute fractional ranks $r_i^{(b)}$ using $w_i^{(b)}$;

2. compute the bootstrap concentration index

$$CI_y^{(b)} = \frac{2}{\bar{y}^{(b)}} \operatorname{Cov}^{(b)}(y_i, r_i);$$

3. estimate the nonlinear model using $w_i^{(b)}$;

4. compute marginal effects and elasticities $\eta_j^{(b)}$;

5. compute covariate concentration indices $CI_{x_j}^{(b)}$;

6. compute contributions $C_j^{(b)} = \eta_j^{(b)} CI_{x_j}^{(b)}$.

This procedure resamples the full estimation pipeline, ensuring that uncertainty in the concentration index, the nonlinear model, the marginal effects, and the covariate concentration indices is propagated into the final standard errors. Bootstrap standard errors are computed from the empirical distribution of $\{C_j^{(b)}\}_{b=1}^{B}$.

Justification

This bootstrap approach is appropriate for three reasons. First, the decomposition is a nonlinear functional of weighted sample moments and regression parameters, for which analytic variance formulas are not available. Second, the use of replicate weights preserves the survey-weighted structure of the data and yields design-consistent variance estimates. Third, resampling the entire decomposition ensures that all sources of sampling variability are incorporated, including uncertainty in the nonlinear model and in the ranking of individuals by socioeconomic status.
